# Personality profile and its association with conversion to neurodegenerative disorders in idiopathic REM sleep behavior disorder

**DOI:** 10.1038/s41531-022-00356-1

**Published:** 2022-07-14

**Authors:** Li Zhou, Steven W. H. Chau, Yaping Liu, Jing Wang, Jihui Zhang, Ngan Yin Chan, Joey W. Y. Chan, Bei Huang, Sijing Chen, Shirley Xin Li, Vincent Chung Tong Mok, Yun Kwok Wing

**Affiliations:** 1 Department of Psychiatry, Faculty of Medicine, The Chinese University of Hong Kong, Shatin, N.T., Hong Kong SAR China; 2 Li Chiu Kong Family Sleep Assessment Unit, Department of Psychiatry, Faculty of Medicine, The Chinese University of Hong Kong, Shatin, Hong Kong SAR China; 3 Guangdong Mental Health Center, Guangdong Provincial People’s Hospital, Guangdong Academy of Medical Sciences, Guangzhou, Guangdong China; 4 Department of Psychology, The University of Hong Kong, Pokfulam, Hong Kong SAR China; 5 The State Key Laboratory of Brain and Cognitive Sciences, The University of Hong Kong, Pokfulam, Hong Kong SAR China; 6 Department of Medicine and Therapeutics, Faculty of Medicine, The Chinese University of Hong Kong, Shatin, Hong Kong SAR China

**Keywords:** Parkinson's disease, Circadian rhythms and sleep

## Abstract

Patients with Parkinson’s disease (PD) were described less extraverted and more neurotic. It remained unclear whether similar personality traits could be found in idiopathic rapid eye movement sleep behavior disorder (iRBD), a prodromal stage of PD, and could predict phenoconversion to neurodegenerative disorders. We aimed to investigate the personality profile and its association with future neurodegenerative phenoconversion in iRBD patients. One hundred and eighty-five video-polysomnography confirmed iRBD patients and 91 age- and sex-matched controls underwent personality assessment using the NEO five-factor inventory, and 171 iRBD patients were followed up. Our results showed that iRBD was marginally negatively associated with extraverted personality trait (B = −0.28, 95% confidence interval (CI) = −0.55, −0.001). During a median follow-up of 5.9 years, 47 iRBD patients (27.5%) had phenoconversion. More neurotic (adjusted hazard ratio (HR) = 2.0, 95% CI = 1.3, 3.1) and less extraverted personality traits (adjusted HR = 0.53, 95% CI = 0.36, 0.77) were associated with an increased risk of phenoconversion in iRBD patients. Our findings suggest that personality profile may be a potential prodromal marker of iRBD.

## Introduction

Idiopathic rapid eye movement sleep (REM) behavior disorder (iRBD) is a distinct parasomnia characterized by recurrent dream-enactment behaviors and REM sleep without atonia (RSWA)^1^. More than 90% of patients with iRBD were found to develop α-synucleinopathies, including Parkinson’s disease (PD), dementia of Lewy bodies (DLB), and multiple system atrophy (MSA) within 15 years^1,2^. Thus, iRBD is currently regarded as a prodromal stage of α-synucleinopathy, and it is important to identify predictors for delineating the neurodegenerative trajectory.

Typical personality traits, characterized by a lower level of extraversion, higher levels of neuroticism and harm-avoidance, and a lack of novelty-seeking, have been reported in PD patients^3,4^. However, it remained unclear whether such personality traits might have already been presented in the prodromal stage of PD. To our knowledge, there were only 3 studies that have investigated personality traits in iRBD patients. While two studies found that patients with iRBD tend to have higher levels of harm-avoidance and neuroticism but lower levels of extraversion and openness when compared with healthy controls^4,5^, the other study did not find any association between personality profile and iRBD^6^. The inconsistent data may be partly contributed by the varying sample size of the studies and whether there was adequate control of major confounding factors, such as psychiatric disorders and mood state of the iRBD patients. In addition, the only study that investigated whether personality traits could predict phenoconversion in iRBD patients did not find any significant association^7^. Nonetheless, the limited sample size of the study (*n* = 46) and presence of some confounding factors may preclude any definitive conclusion^7^. Therefore, more studies are needed to investigate the longitudinal predictive performance of personality traits in iRBD regarding phenoconversion. Our study aimed to investigate the relationship between personality traits and iRBD, and to explore the prognostic implication of personality traits in predicting future phenoconversion.

## Results

### Cross-sectional case-control study

#### Demographics and personality traits of all subjects at baseline

**Fig. 1:**
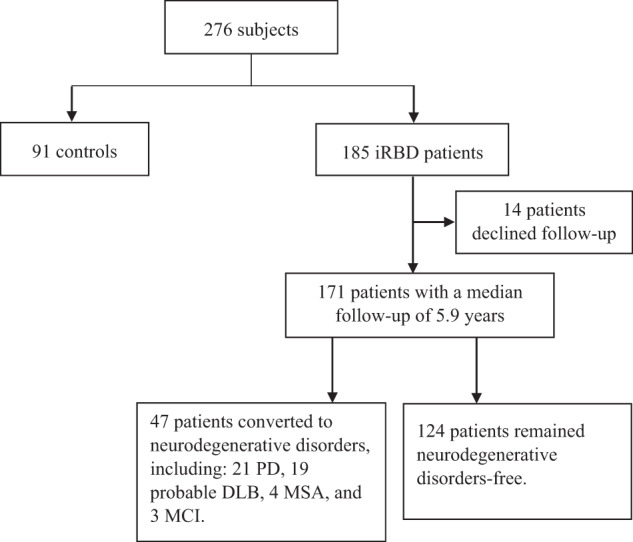
Flowchart of study. DLB, Dementia with Lewy bodies; iRBD, idiopathic rapid eye movement sleep behavior disorder; MCI, mild cognitive impairment; MSA, multiple system atrophy; PD, Parkinson’s disease.

A total of 185 patients with iRBD and 91 controls were recruited at baseline (Fig. 1: flowchart of the study). Demographic characteristics and personality traits of all subjects were shown in Table 1. The baseline age and sex were comparable between iRBD patients and controls (median [interquartile range, IQR] of age: 64.0 [12.0] vs. 66.0 [13.0] years, Z score = −0.74, *p* = 0.46, *r* = 0.045; male sex: 70.3% vs. 65.9%, *χ*^2^ = 0.54, *p* = 0.47, *ϕ* = 0.044). Although iRBD patients had a marginally higher level of education (20.0% vs. 10.2%, *χ*^2^ = 4.1, *p* = 0.044, *P*_adjusted_ = 0.087, *ϕ* = 0.12), they had a relatively lower score of education-adjusted Hong Kong Montreal Cognitive Assessment (HK-MoCA) than controls (median [IQR]: 25.0 [3.0] vs. 26.0 [3.0], Z score = −2.6, *p* = 0.011, *P*_adjusted_ = 0.037, *r* = 0.15). The total scores of the short form of the Beck Depression Inventory (BDI-13) (median [IQR]: 5.0 [8.0] vs. 3.0 [7.0], Z score = −3.7, *p* < 0.001, *P*_adjusted_ = 0.002, *r* = 0.23) and RBD Questionnaire-Hong Kong (RBDQ-HK) (median [IQR]: 43.0 [24.0] vs. 5.0 [10.0], Z score = −12.9, *p* < 0.001, *P*_adjusted_ < 0.001, *r* = 0.78) were higher in iRBD patients when compared with controls. In addition, iRBD patients had a higher percentage of current smokers (15.3% vs. 4.5%, *χ*^2^ = 6.7, *p* = 0.009, *P*_adjusted_ = 0.036, *ϕ* = 0.16), marginally lower percentages of non-smokers (74.9% vs. 85.4%, *χ*^2^ = 3.9, *p* = 0.048, *P*_adjusted_ = 0.087, *ϕ* = −0.12) and non-alcohol drinkers (53.0% vs. 65.9%, *χ*^2^ = 4.0, *p* = 0.045, *P*_adjusted_ = 0.087, *ϕ* = −0.12), and marginally higher percentage of lifetime or current psychiatric disorders than controls (39.5% vs. 26.4%, *χ*^2^ = 4.6, *p* = 0.032, *P*_adjusted_ = 0.083, *ϕ* = 0.13) (Table 1).

**Table 1:** Demographic characteristics, clinical features, and personality traits of iRBD patients and controls at baseline.

	Patients with iRBD (*n* = 185)	Controls (*n* = 91)	*p*	*P*_adjusted_	Standardized effect size
Age, years	64.0 [12.0]	66.0 [13.0]	0.46**	0.52	0.045^b^
Male	130 (70.3)	60 (65.9)	0.47*	0.52	0.044^c^
Education, tertiary or above	37 (20.0)	9 (10.2)	0.044*	0.087	0.12^c^
BMI, kg/m^2^	24.4 ± 3.5 (*n* = 182)	25.0 ± 3.7 (*n* = 88)	0.21*	0.30	0.17^d^
Alcohol drinking					
Never	96 (53.0)	58 (65.9)	0.045*	0.087	−0.12^c^
Former	24 (13.3)	5 (5.7)	0.06*	0.10	0.12^c^
Current	61 (33.7)	25 (28.4)	0.38*	0.51	0.05^c^
Smoking					
Never	137 (74.9)	76 (85.4)	0.048*	0.087	−0.12^c^
Former	18 (9.8)	9 (10.1)	0.94*	0.94	−0.004^c^
Current	28 (15.3)	4 (4.5)	0.009*	**0.036**	0.16^c^
BDI-13, total score	5.0 [8.0] (*n* = 184)	3.0 [7.0]	<0.001**	**0.002**	0.23^b^
ESS, total score	8.0 [9.0] (*n* = 182)	8.0 [9.0] (*n* = 90)	0.56**	0.59	0.036^b^
RBDQ-HK, total score	43.0 [24.0]	5.0 [10.0]	<0.001**	**<0.001**	0.78^b^
HK-MoCA, total score^a^	25.0 [3.0]	26.0 [3.0]	0.011**	**0.037**	0.15^b^
Comorbidity of psychiatric disorders	73 (39.5)	24 (26.4)	0.032*	0.083	0.13^c^
*Big Five personality inventory*					
Neuroticism, score	21.0 [10.0]	17.0 [10.0]	0.001**	**0.007**	0.19^b^
Extraversion, score	23.2 ± 5.8	25.3 ± 5.7	0.006*	**0.03**	0.36^d^
Openness, score	23.0 [5.1]	24.0 [5.4]	0.46**	0.52	0.045^b^
Agreeableness, score	30.0 [5.0]	32.0 [5.6]	0.15**	0.23	0.087^b^
Conscientiousness, score	31.0 [6.0]	32.0 [6.6]	0.033**	0.083	0.13^b^

Data are shown as mean ± standard deviation, *n* (%), or median [interquartile range].

*BDI-13* the short form of the Beck Depression Inventory; *BMI* body mass index, *ESS* the Epworth Sleepiness Scale, *HK-MoCA* Hong Kong Montreal Cognitive Assessment, *iRBD* idiopathic rapid eye movement sleep behavior disorder, *RBDQ-HK* rapid eye movement sleep behavior disorder questionnaire‐Hong Kong.

The Benjamini–Hochberg procedure was used to adjust *p* values. In bold indicated *p* value <0.05.

^a^The score of HK-MoCA was adjusted for education level.

Effect size^b^ represents *r*, Effect size^c^ represents *ϕ*, and Effect size^d^ represents Hedge’s *g*.

*P* value* was estimated with independent *t*-test or Chi-square test.

*P* value** was estimated with nonparametric Mann–Whitney U test.

When compared with controls, iRBD patients had a higher score in neuroticism (median [IQR]: 21.0 [10.0] vs. 17.0 [10.0], Z score = −3.2, *p* = 0.001, *P*_adjusted_ = 0.007, *r* = 0.19), a lower score of extraversion (mean ± standard deviation (SD): 23.2 ± 5.8 vs. 25.3 ± 5.7, *t* value = 2.8, degree of freedom (df) = 274, *p* = 0.006, *P*_adjusted_ = 0.03, Hedge’s *g* = 0.36), and a marginally lower score of conscientiousness (median [IQR]: 31.0 [6.0] vs. 32.0 [6.6], Z score = −2.1, *p* = 0.033, *P*_adjusted_ = 0.083, *r* = 0.13). The scores of openness and agreeableness were comparable between iRBD patients and controls (Table 1).

#### Correlations between iRBD and personality traits

iRBD was positively correlated with the score in neuroticism (*β* = 0.20; *t* = 3.3, B, 0.42; 95% CI, 0.17 to 0.66; *p* = 0.001; *P*_adjusted_ = 0.01; post hoc power = 0.91) and negatively correlated with the score in extraversion (*β* = −0.17; *t* = 2.8; B, −0.35; 95% CI, −0.60 to −0.10; *p* = 0.006; *P*_adjusted_ = 0.03; post hoc power = 0.79) in the unadjusted model. In addition, iRBD was marginally negatively correlated with the score in conscientiousness (*β* = −0.13; *t* = −2.2; B, −0.28; 95% CI, −0.53 to −0.03; *p* = 0.031; *P*_adjusted_ = 0.10; post hoc power = 0.58). There was no significant correlation between iRBD and other two personality traits in the unadjusted model. In the adjusted model, iRBD was only marginally negatively correlated with the score of extraversion (*β* = −0.13; *t* = −2.0; B, −0.28; 95% CI, −0.55 to -0.001; *p* = 0.049; *P*_adjusted_ = 0.12; post hoc power = 0.50). There was no significant correlation of iRBD with other four personality traits in the adjusted models (Table 2).

**Table 2:** Correlations between iRBD and personality traits.

	Unadjusted model							Adjusted model^a^						
	*R*^2^	*β*	*t*	B (95% CI)	*p*	*P*_adjusted_	Post hoc power	*R*^2^	*β*	*t*	B (95% CI)	*p*	*P*_adjusted_	Post hoc power
N	0.04	0.20	3.3	0.42 (0.17, 0.66)	**0.001**	**0.01**	0.91	0.47	0.06	1.1	0.13 (−0.10, 0.36)	0.26	0.37	0.20
E	0.03	−0.17	−2.8	−0.35 (−0.60, −0.10)	**0.006**	**0.03**	0.79	0.25	−0.13	−2.0	−0.28 (−0.55, −0.001)	**0.049**	0.12	0.50
O	<0.001	0.001	0.01	0.002 (−0.25, 0.25)	0.99	0.99	0.05	0.11	−0.07	−1.0	−0.15 (−0.45, 0.14)	0.32	0.40	0.17
A	0.007	−0.08	−1.4	−0.18 (−0.43, 0.08)	0.17	0.28	0.28	0.09	−0.02	−0.3	−0.20 (−1.6, 1.2)	0.78	0.87	0.06
C	0.02	−0.13	−2.2	−0.28 (−0.53, −0.03)	**0.031**	0.10	0.58	0.11	−0.13	−1.8	−1.3 (−2.7, 0.13)	0.074	0.15	0.43

The Benjamini–Hochberg procedure was used to adjust *p* values. In bold indicated *p* value <0.05.

*A* agreeableness, *BDI-13* the short form of the Beck Depression Inventory, *C* conscientiousness, *E* extraversion, *HK-MoCA* Hong Kong Montreal Cognitive Assessment, *iRBD* idiopathic rapid eye movement sleep behavior disorder, *N* neuroticism, *O* openness.

^a^Model was adjusted for age, sex, education-adjusted HK-MoCA, education level, BDI-13, psychiatric disorders, and psychiatric disorders*group.

#### Follow-up study

A total of 171 patients were successfully followed up with a median of 5.9 years. Among them, 47 iRBD patients (27.5%) developed neurodegenerative disorders (convertors), including 21 PD, 19 probable DLB, 4 MSA, and 3 mild cognitive impairment (MCI) (Table 3).

**Table 3:** Demographics, neurodegenerative biomarkers, and personality traits between non-convertors and convertors at baseline.

	Non-convertors (*n* = 124)	Convertors (*n* = 47)	*p*	*P*_adjusted_	Standardized effect size
Age at baseline, years	62.4 ± 9.9	69.6 ± 6.8	<0.001*	**<0.001**	0.79^d^
Age at onset of RBD symptoms, years	58.0 [11.0] (*n* = 123)	61.5 [9.0]	0.003**	**0.02**	0.23^b^
Duration of RBD symptoms at baseline, years	5.0 [4.9] (*n* = 123)	5.2 [6.0]	0.086**	0.20	0.13^b^
Duration of follow-up, years	6.3 [4.5]	4.9 [5.5]	0.001**	**0.009**	0.25^b^
Male	85 (68.5)	37 (78.7)	0.19*	0.31	0.10^c^
Education, tertiary or above	24 (19.4)	8 (17.0)	0.73*	0.80	−0.027^c^
Alcohol drinking					
Never	59 (48.4)	29 (64.4)	0.065*	0.19	0.14^c^
Former	14 (11.5)	6 (13.3)	0.74*	0.80	0.025^c^
Current	49 (40.2)	10 (22.2)	0.031*	0.13	−0.17^c^
Smoking					
Never	90 (72.6)	34 (75.6)	0.70*	0.80	0.03^c^
Former	9 (7.3)	8 (17.8)	0.044*	0.14	0.16^c^
Current	25 (20.2)	3 (6.7)	0.037*	0.14	−0.16^c^
RBDQ-HK, total score	42.0 ± 17.5	43.8 ± 17.8	0.56*	0.77	0.10^d^
BDI-13, total score	5.0 [9.0] (*n* = 123)	5.0 [5.0]	0.62**	0.80	0.04^b^
ESS, total score	9.0 [9.0] (*n* = 123)	8.0 [7.0] (*n* = 45)	0.12**	0.23	0.12^b^
UPDRS-III, total score	2.0 [4.0] (*n* = 75)	4.5 [4.8] (*n* = 20)	0.068**	0.18	0.19^b^
OIT, correct score	2.0 [3.0] (*n* = 117)	1.0 [2.0] (*n* = 41)	0.026**	0.13	0.18^b^
HK-MoCA, total score^a^	26.0 [3.0]	25.0 [2.0]	0.13**	0.23	0.12^b^
The comorbidity of psychiatric disorders	59 (47.6)	8 (17.0)	<0.001*	**0.003**	−0.28^c^
*Polysomnographic features*					
Tonic chin electromyography level, %	10.2 [25.6] (*n* = 114)	22.5 [40.7] (*n* = 40)	0.096**	0.21	0.13^b^
Phasic chin electromyography level, %	13.7 [10.0] (*n* = 114)	12.5 [13.5] (*n* = 40)	0.99**	0.99	0.002^b^
*Big Five personality inventory*					
Neuroticism, score	21.3 ± 7.7	20.5 ± 5.4	0.45*	0.65	0.11^d^
Extraversion, score	23.1 ± 5.9	23.4 ± 5.5	0.79*	0.82	0.05^d^
Openness, score	24.0 [5.2]	23.0 [5.0]	0.13**	0.23	0.12^b^
Agreeableness, score	31.0 ± 4.1	30.2 ± 3.8	0.24*	0.37	0.20^d^
Conscientiousness, score	31.1 ± 4.7	30.7 ± 4.5	0.67*	0.80	0.09^d^
*Neurodegenerative disorders*					
PD	–	21 (44.7)	NA	NA	NA
Probable DLB	–	19 (40.4)	NA	NA	NA
MSA	–	4 (8.5)	NA	NA	NA
MCI	–	3 (6.4)	NA	NA	NA

Data are shown as mean ± standard deviation, *n* (%), or median [interquartile range].

The Benjamini–Hochberg procedure was used to adjust *p* values. In bold indicated *p* value <0.05.

*BDI-13* the short form of the Beck Depression Inventory, *DLB* Dementia with Lewy bodies, *ESS* the Epworth Sleepiness Scale, *HK-MoCA* Hong Kong Montreal Cognitive Assessment, *iRBD* idiopathic rapid eye movement sleep behavior disorder, *MCI* mild cognitive impairment, *MSA* multiple system atrophy, *NA* not applicable, *OIT* Olfactory Identification test, *PD* Parkinson’s disease, *RBDQ-HK* rapid eye movement sleep behavior disorder questionnaire‐Hong Kong, *UPDRS-III* Unified Parkinson’s Disease Rating Scale-part III.

^a^The score of HK-MoCA was adjusted for education level.

Effect size^b^ represents *r*, Effect size^c^ represents *ϕ*, Effect size^d^ represents Hedge’s g.

*P* value* was estimated with independent *t*-test or Chi-square test.

*P* value** was estimated with nonparametric Mann–Whitney U test.

Table 3 shows the baseline clinical features and personality traits of iRBD patients. Convertors were older at baseline (mean ± SD: 69.6 ± 6.8 vs. 62.4 ± 9.9 years, *t* value = 4.6, df = 169, *p* < 0.001, *P*_adjusted_ < 0.001, Hedge’s *g* = 0.79), had later onset age of RBD symptoms (median [IQR]: 61.5 [9.0] vs. 58.0 [11.0] years, Z score = −3.0, *p* = 0.003, *P*_adjusted_ = 0.02, *r* = 0.23), and had a shorter follow-up duration (median [IQR]: 4.9 [5.5] vs. 6.3 [4.5] years, Z score = −3.3, *p* = 0.001, *P*_adjusted_ = 0.009, *r* = 0.25) compared with non-convertors (neurodegenerative disease-free patients during follow-up). Convertors had a marginally higher percentage of former smokers (17.8% vs. 7.3%, *χ*^2^ = 4.0, *p* = 0.044, *P*_adjusted_ = 0.14, *ϕ* = 0.16), but a marginally lower percentage of current alcohol drinkers (22.2% vs. 40.2%, *χ*^2^ = 4.6, *p* = 0.031, *P*_adjusted_ = 0.13, *ϕ* = −0.17) and current smokers (6.7% vs. 20.2%, *χ*^2^ = 4.4, *p* = 0.037, *P*_adjusted_ = 0.14, *ϕ* = −0.16) when compared with non-convertors. In addition, Convertors had a lower percentage of comorbidity of psychiatric disorders than non-convertors (17.0% vs. 47.6%, *χ*^2^ = 13.4, *p* < 0.001, *P*_adjusted_ = 0.003, *ϕ* = −0.28). There was a marginally lower score of olfactory identification test in convertors as compared with non-convertors (1.0 [2.0] vs. 2.0 [3.0], Z score = −2.2, *p* = 0.026, *P*_adjusted_ = 0.13, *ϕ* = 0.18). There was no statistically significant difference in sex, duration of RBD, education level, phasic and tonic electromyography (EMG) level, scores of the RBDQ-HK, BDI-13, Epworth Sleepiness Scale, Unified Parkinson’s Disease Rating Scale-part III, education-adjusted HK-MoCA, and personality traits between the two groups.

#### Correlations of personality traits with conversion risk in iRBD patients

In the unadjusted Cox regression model, openness was a marginally significant predictor of conversion (hazard ratio (HR) = 0.73; 95% CI = 0.54 to 0.98; *p* = 0.035; *P*_adjusted_ = 0.12; post hoc power = 0.54). In the adjusted Cox regression models, both neuroticism and extraversion were statistically significant predictors of conversion. An increase of one SD score from mean in neuroticism was associated with an 100% increased risk of conversion (adjusted HR, 2.0; 95% CI, 1.3 to 3.1; *p* = 0.002; *P*_adjusted_ = 0.01; post hoc power = 0.86), while an increase of one SD score from mean in extraversion was associated with a 47% decreased risk (adjusted HR, 0.53; 95% CI, 0.36 to 0.77; *p* < 0.001; *P*_adjusted_ = 0.009; post hoc power = 0.79) (Table 4).

**Table 4:** Risk of neurodegenerative disorders by personality traits in iRBD patients.

	Unadjusted model					Adjusted model^a^				
	*R*^2^	HR (95% CI)	*p*	*P*_adjusted_	Post hoc power	*R*^2^	HR (95% CI)	*p*	*P*_adjusted_	Post hoc power
N	0.006	0.92 (0.68, 1.2)	0.59	0.66	0.08	0.59	2.0 (1.3, 3.1)	**0.002**	**0.01**	0.86
E	0.003	0.94 (0.71, 1.3)	0.69	0.69	0.06	0.60	0.53 (0.36, 0.77)	**<0.001**	**0.009**	0.79
O	0.093	0.73 (0.54, 0.98)	**0.035**	0.12	0.54	0.51	0.84 (0.60, 1.2)	0.31	0.39	0.13
A	0.035	0.81 (0.59, 1.1)	0.19	0.32	0.29	0.52	0.77 (0.53, 1.1)	0.16	0.32	0.24
C	0.026	0.84 (0.62, 1.1)	0.26	0.37	0.22	0.53	0.74 (0.52, 1.1)	0.10	0.25	0.29

^a^Model was adjusted for age at baseline, sex, education-adjusted HK-MoCA, psychiatric disorders, and psychiatric disorders*personality traits.

The Benjamini–Hochberg procedure was used to adjust *p* values. In bold indicated *p* value <0.05.

*CI* confidence interval, *HK-MoCA* Hong Kong Montreal Cognitive Assessment, *HR* hazard ratio, *iRBD* idiopathic rapid eye movement sleep behavior disorder.

## Discussion

The present study found that patients with iRBD were marginally associated with less extraverted personality trait. Furthermore, more neurotic and less extraverted personality traits were associated with an increased risk of phenoconversion in iRBD patients. These findings suggested that personality profile might potentially serve as a prodromal marker of neurodegeneration in iRBD.

Our case-control study found that iRBD patients were marginally less extraverted when compared with age- and sex-matched controls, which is compatible with previous study^4^. This pattern of personality profile has been observed in the patients with PD^4^. Although a previous study reported that iRBD patients, apart from being less extraverted, were also more neurotic and less open to experiences^4^. However, our study showed that there was no significant association between neuroticism and iRBD after further controlling of the psychiatric comorbidities and other confounders. Interestingly, the previous study found that the presence of neurotic personality trait was more likely related to those PD subjects who were comorbid with depression^8^. Thus, it is possible that neuroticism may be more related to iRBD patients comorbid with psychiatric disorders.

Our longitudinal study has identified that more neurotic and less extraverted personality trait would confer a higher risk of phenoconversion in iRBD patients. Although a previous study did not find any prediction of personality trait to future risk of neurodegeneration in iRBD patients^7^, our current study with a larger sample size was able to find an association between the personality profile and phenoconversion in iRBD patients.

The mechanism underlying the associations of personality traits with iRBD and PD remained unclear. There may be two possible explanations that are not mutually exclusive to each other. First, a ‘premorbid’ personality trait might predispose vulnerable individuals to developing α-synucleinopathy neurodegeneration. Our study showed that a lower level of extraversion in the personality trait was already found at the prodromal stage of α-synucleinopathies (i.e., iRBD stage). Although a recent meta-analysis suggested that neuroticism was related to an increased risk of PD, it did not take into account of the potential confounding effect of psychiatric disorders, which have been linked to both neuroticism and PD^9^. In this regard, a recent Mendelian randomization study did not find the causal effect of neuroticism on PD^10^, and our study also found that there was no association between neuroticism and iRBD. Second, the personality trait might be an early manifestation of α-synucleinopathy neurodegeneration. iRBD is regarded as the most robust prodromal marker of α-synucleinopathy neurodegeneration, as its occurrence was postulated to be related to the involvement of locus subcoeruleus nucleus in human^1^. The subtle involvement and dysfunction of norepinephrine-producing nucleus (locus coeruleus) and serotonergic neurons (raphe nuclei) at the early stage may influence personality development^11,12^.

Moreover, there might be a complex relationship between personality trait and α-synucleinopathy neurodegeneration. For example, less extraverted individuals might predispose themselves to more stressful life events. Sustained stress could lead to the over-activation of hypothalamic–pituitary–adrenal axis with excessive glucocorticoids and neuroinflammatory reactions, resulting in the accelerated death of neurons^13^. On the other hand, there might be a shared genetic correlation between personality traits and α-synucleinopathy neurodegeneration, as there was a reported correlation between a lower level of extraversion and dopamine-related genes, such as D4 dopamine receptor^14^.

In addition, our study found that open personality trait was marginally associated with a lower risk of phenoconversion initially (without any adjustment). However, the predictive risk of openness became non-significant after adjusting for multiple variables. The explanation for this result may be that openness trait might be affected by other variables in iRBD. Previous studies suggested that older age and poor cognition could predict future phenoconversion in iRBD patients, which are also tightly related to open personality trait^15–18^. Thus, age and cognition might modulate the effect of openness on future phenoconversion. However, further studies are needed to confirm this postulation.

There are some limitations in our study. First, there were relatively fewer controls in the study. The second limitation was that as a clinic-based study, all the diagnosis of neurodegenerative diseases did not have pathological confirmation. Third, the causal relationship between personality profile and iRBD could not be inferred in our cross-sectional nature of study. Fourth, limited sample of phenoconvertors in our study could not take into account of the interdependencies between different personality traits. Further study with a larger sample size may address this limitation. Last, our findings may not be extrapolated to other RBD cohorts due to the ethnic/cultural effect on personality trait. Further prospective studies are needed to explore the progressive evolution of personality change, and preferably starting from the prodromal RBD stage^19^.

In summary, our study showed that there was a possible association between iRBD and a lower level of extraversion. A personality profile of high neuroticism and low extraversion was associated with an increased risk of developing neurodegenerative disorders in iRBD patients. Our study suggests that personality trait might serve as a potential prodromal marker of α-synucleinopathy-related neurodegenerative disorders.

## Methods

### Study design and subject recruitment

The present study consisted of a cross-sectional case-control study that examined the relationship between personality profile and iRBD and a longitudinal follow-up study that investigated whether any particular personality trait could predict future phenoconversion in iRBD patients.

In the case-control study, all subjects were consecutively recruited from June 2009 to June 2017. iRBD patients were recruited from Li Chiu Kong Family Sleep Assessment Unit, while controls were recruited from the community (45%) and Sleep Assessment Unit (55%). Since the study spanned over 2009–2017, the diagnostic criteria of iRBD were based on the second and third edition of International Classification of Sleep Disorder (before and after 2014, respectively)^20,21^. The inclusion criteria for patients were: (1) a diagnosis of RBD as confirmed by video-polysomnography (v-PSG). In summary, patients should present with a history of repeated dream-enactment behaviors or behaviors observed by v-PSG assessment during REM sleep and RSWA. In addition, their RBD symptoms were not better explained by other reasons, such as other sleep disorders or medications. (2) Absence of any neurodegenerative disorder. (3) Absence of narcolepsy. The inclusion criteria for controls were: (1) Age- and sex-matched with iRBD patients. (2) Absence of RBD. (3) Absence of parkinsonism based on the clinical examination and neurocognitive impairment measured by the HK-MoCA (total score <22)^22^.

In the longitudinal study, iRBD patients were followed up in our sleep clinic. The presence of neurodegenerative disorders, including PD, probable DLB, MSA, and MCI, were assessed as based on the standard diagnostic criteria^23–26^. All diagnosis and censoring event including death were documented and collected in the electronic health care database system (Clinical Management System) of the public health care system in Hong Kong.

This study was conducted in accordance with the Declaration of Helsinki and approved by the Joint Chinese University of Hong Kong-New Territories East Cluster Clinical Research Ethics Committee (2008.478 and 2017.129). All subjects gave their written informed consent for collecting their information and clinical records.

### Measurements

#### Questionnaire and clinical assessment

Baseline sociodemographic characteristics, including age, sex, education, alcohol use, smoking, and body mass index, were collected by a self-reported questionnaire. The RBDQ-HK was employed to assess the severity of RBD symptoms^27^. The BDI-13 was used to measure current depressive symptoms^28^. Some earlier cases were measured by Cantonese version of Mini-Mental State Examination and their scores were converted into HK-MoCA score^29,30^. The Epworth Sleepiness Scale was employed to measure excessive daytime sleepiness^24^. The Unified Parkinson’s Disease Rating Scale-part III was used to evaluate motor function^31^. The locally validated olfactory identification test was applied to measure olfactory function^32^.

All diagnosis of lifetime or current psychiatric disorders were clinically diagnosed and retrieved from the Clinical Management System, which is a computerized medical system operated in public hospitals and covers over 95% of healthcare provision in Hong Kong^33^.

#### Personality profile assessment

The Chinese version of NEO-FFI was used to assess the personality profile^34^. The NEO-FFI consists of 60 items on a 5-point Likert-scale for agreement (0 = strongly disagree and 4 = strongly agree)^35^. Five personality dimensions were generated based on sub-scale items: neuroticism (N), extraversion (E), openness (O), agreeableness (A), and conscientiousness (C). The Cronbach’s α coefficients for internal consistency of the Chinese version were 0.82, 0.75, 0.63, 0.72, and 0.81 for N, E, O, A, and C, respectively^34^. In this study, the Cronbach’s α coefficients for N, E, O, A, and C were 0.85, 0.74, 0.57, 0.62, and 0.69, respectively. Raw scores and transformed Z scores were used in the analysis.

#### V-PSG assessment and EMG scoring

The detailed v-PSG protocol and EMG activity scoring method during REM sleep were previously reported^23^. EMG activity of chin muscles was scored by an experienced and registered polysomnographic technologist who was blind to the diagnosis. EMG artifacts associated with arousal or respiratory events were excluded. A 30-s tonic epoch was defined when there was an elevation of EMG amplitude at least twice the background amplitude lasting >15 s. Phasic EMG activity, defined by any EMG burst exceeding 4 times the background amplitude that lasted 0.1–5 s, was scored based on 3-s mini-epoch. The time of phasic mini-epoch or tonic epoch over the whole REM period represented the phasic or tonic EMG level, respectively.

### Statistical analysis

We used the Shapiro-Wilk test and the Normal Q-Q plots to test the normality of continuous variables and linear regression model residuals. Continuous data were shown as mean ± SD or median [IQR] and were compared using independent *t*-test or nonparametric Mann–Whitney U test. Categorical variables were shown as numbers (percentage) and were compared by Chi-squared test or Fisher exact test where appropriate. The standardized effect sizes, including Hedge’s *g*, *r*, and Phi (*ϕ*), were calculated for independent *t*-test, nonparametric Mann–Whitney U test, and Chi-squared test, respectively. Linear regression models were used to assess the associations between personality traits (dependent variable) and iRBD status (independent variable) and assumptions were checked. The raw scores of personality traits were transformed to standard scores (Z scores) before entering the models (one unit of Z score represents one standard deviation from mean). To control the confounding effect of psychiatric disorders, psychiatric disorders and interaction term of group*psychiatric disorders were adjusted in the linear regression models. Other covariates included age, sex, education level, BDI-13, and education-adjusted HK-MoCA.

The Cox proportional hazards regression models with “survival” package in R (NEO-FFI Z scores were set as predictor) were performed to calculate the HR and the 95% CI for developing neurodegenerative disorders and the assumption of proportional hazards in Cox models was tested. The endpoint event was the diagnosis of any neurodegenerative disorders (PD, probable DLB, MSA, or MCI). Censoring date was defined as date of death or date of last visit. Due to the limited number of converted cases, we combined all the neurodegenerative disorders in the analysis of the association between personality trait and the risk of neurodegeneration in iRBD patients. To control the confounding effect of psychiatric disorders, psychiatric disorders and interaction term of personality traits*psychiatric disorders were adjusted in the Cox regression models. Other covariates included age, sex, and HK-MoCA. The R^2^ and post hoc power of Cox regression models were calculated with the “CoxR2” and “powerSurvEpi” packages in R, respectively.

All statistical analyses were performed on SPSS version 26 (IBM, Armonk, NY) and R project (version 4.1.0). In consideration of multiple comparisons, *p* values were adjusted using Benjamini–Hochberg procedure. A two-tailed *p* value < 0.05 was considered statistically significant.

## Data Availability

Data that support the findings of this study are available with a reasonable request from the Department of Psychiatry, Faculty of Medicine, The Chinese University of Hong Kong, via the corresponding author.
